# *Fusobacterium nucleatum* Enhances Intestinal Adaptation of *Vibrio cholerae* via Interspecies Biofilm Formation

**DOI:** 10.3390/microorganisms14010211

**Published:** 2026-01-16

**Authors:** Guozhong Chen, Jiamin Chen, Xiangfeng Wang, Dingming Guo, Zhi Liu

**Affiliations:** 1 Department of Biotechnology, College of Life Science and Technology, Huazhong University of Science and Technology, Wuhan 430074, China; 2023612479@hust.edu.cn (J.C.); d202080696@hust.edu.cn (X.W.); D202080693@hust.edu.cn (D.G.); 2 State Key Laboratory of Microbial Technology, Shandong University, Qingdao 266237, China

**Keywords:** *Vibrio cholerae*, biofilm, *Fusobacterium nucleatum*, coaggregation

## Abstract

Biofilm formation represents a key survival strategy employed by *Vibrio cholerae* to adapt to the complex intestinal environment of the host. While most previous studies on *V. cholerae* biofilms have focused on genetic regulation and monospecies cultures, its ability to form dual-species biofilms with other intestinal pathogens is still poorly understood. In this study, using samples from both cholera patients and healthy individuals, *Fusobacterium nucleatum* was identified as a bacterium capable of co-aggregating with *V. cholerae*. Untargeted metabolomic analysis revealed that *F. nucleatum*-derived metabolites, specifically 6-hypoxanthine, enhance biofilm formation in *V. cholerae*. Further validation confirmed that these *F. nucleatum*-derived metabolites upregulate the biofilm-associated regulatory gene *vpsT*. In an adult mouse model, co-infection with *F. nucleatum* and *V. cholerae* significantly enhanced the intestinal adaptability of *V. cholerae* compared to infection with *V. cholerae* alone. Together, these findings elucidate the mechanism enabling the co-infection of *F. nucleatum* and *V. cholerae* in the host intestine, thereby shedding new light on how other pathogenic bacteria can assist in *V. cholerae* infection.

## 1. Introduction

*Vibrio cholerae* is a Gram-negative pathogen responsible for cholera in humans. It is transmitted through the ingestion of contaminated water or food, colonizes the small intestine, and secretes cholera toxin (CT), leading to severe watery diarrhea and dehydration [1]. Cholera, caused by *V. cholerae*, remains a major global public health concern. The World Health Organization (WHO) has reported a large number of cholera cases or outbreaks, from 44 countries in 2022 to 45 countries in 2023, and shown an increase in both the number of people who fell sick and died from the Cholera. Also, reported cholera cases rose by 5% and deaths by 50% in 2024 compared to 2023. It is responding with urgency to reduce deaths and outbreaks in countries around the world [2].

In recent years, the role of the gut microbiota in modulating pathogenic infections has gained increasing attention [3,4,5]. Studies indicated that commensal or pathogenic bacteria can influence the colonization efficiency of pathogens through metabolic cross-talk, spatial competition, or biofilm formation [6].

Biofilm formations, a critical factor complicating cholera management, are the dominant survival strategy for bacteria to tolerate a broad spectrum of intestinal stressors or aquatic reservoirs [6]. Our current understanding of *V. cholerae* biofilm formation, however, is largely derived from studies of single-species systems. Evidence from recent research indicates that flagellar-motility Δ*fliC* and Δ*motA* mutants of *Escherichia coli* play an important role in the coaggregation formation between *V. cholerae* and *E. coli* [7]. In addition, Abriat et al. measured the mechanical properties of the pellicle formed at the air-liquid interface and compared the unique viscoelastic profile for each single-species organism to the dual-species biofilms between *V. cholerae* and *E. coli* [8].

*Fusobacterium nucleatum*, Gram-negative anaerobic bacterium that acts as a periodontal pathogen, being an important factor in linking Gram-positive and Gram-negative bacteria within oral biofilms. Beyond its role in oral disease, it has been extensively studied in the context of colorectal cancer [9]. In the gut, *F. nucleatum* acts as a potent coaggregation pathogen, employing a diverse array of adhesins to facilitate interspecies interactions and drive multispecies biofilm formation. The RadD adhesin plays a central role by mediating arginine-inhibitable adherence, critical for coaggregation with species such as *Streptococcus mutans* [10,11] and *Clostridioides difficile* [12], thereby promoting biofilm development in environments like intestinal mucus. Other surface molecules, including FomA, significantly enhance biofilm formation and metabolic activities in partnership with pathogens such as *Porphyromonas gingivalis* [13]. Additionally, regulatory proteins like CmpA [14], Aid1 [15], FAD-I [16], and Fap2 [17] modulate the specificity and expression of RadD, fine-tuning the binding capacity of Fusobacterium. Meanwhile, FadA mediates biofilm-associated antibiotic tolerance during interactions with *Pseudomonas aeruginosa* [18,19]. In addition, it has been reported that *F. nucleatum* can form dual-species biofilms with several other bacteria, including *Parvimonas micra* [20], *Streptococcus gordonii* [11], *Limosilactobacillus reuteri* [21], *Prevotella* species [22], and *Capnocytophaga ochracea* [23]. These mechanisms collectively enable *F. nucleatum* to function as a keystone orchestrator within polymicrobial communities.

Polymicrobial interactions are a major contributor to most clinical infections [24]. These infections, which often originate in healthcare settings or involve medical devices, arise from complex multi-species communities. A significant challenge in treating these infections stems from biofilms formed through co-aggregation, which enhance bacterial persistence and therapeutic resistance [24]. Current research focuses on interventions targeting various stages of the biofilm lifecycle or employing strategies like phage therapy to disrupt these structures [25,26]. Beyond oral health, *F*. *nucleatum* is now implicated in a spectrum of systemic conditions, including atherosclerosis, rheumatoid arthritis, inflammatory bowel disease (IBD), and adverse pregnancy outcomes [26,27]. This underscores its role as a common pathogen capable of influencing diverse disease processes. Given that *F. nucleatum* can colonize the gut, there exists a plausible spatial opportunity for interaction with other intestinal pathogens like *V*. *cholerae* [3,26]. However, whether and how *F. nucleatum* directly interacts with *V. cholerae*, particularly through mechanisms like joint biofilm formation to enhance intestinal colonization and adaptation, is still unknown and requires elucidation.

In this study, analysis of a cholera cohort revealed a significant difference in the abundance of *F. nucleatum* between stool samples from cholera patients and healthy controls. Consistent with this, comparison of isolates from our microbial bank also showed a marked difference in *F. nucleatum* abundance between the two groups. Further investigation demonstrated that while the metabolite supernatant of *F. nucleatum* inhibited the growth of *V. cholerae*, *F. nucleatum* enhanced coaggregation with the *V. cholerae* and dual-species biofilm formation. Mechanistically, untargeted metabolomic analysis revealed that metabolites derived from *F. nucleatum*, specifically 6-hypoxanthine, promote biofilm formation in *V. cholerae* by upregulating the expression of the biofilm-associated regulatory gene *vpsT*. Subsequent validation in an adult mouse infection model confirmed that *F. nucleatum* promotes the gut adaptability of *V. cholerae*. Together, these findings elucidate that *F. nucleatum* enhances intestinal adaptation of *V. cholerae* via interspecies biofilm formation. This provides a mechanistic basis for how interactions between pathogenic bacteria can affect host health.

## 2. Materials and Methods

### 2.1. Differential Abundance of Fusobacterium Genus in the Cholera Cohort

The six publicly available gut metagenomic datasets from cohort-based studies on cholera were accessed, with accession numbers PRJNA352220 [28], PRJNA668607 [29], PRJEB30604, PRJNA1035800, PRJEB11419, and PRJNA976726 [30] in the NCBI database. The raw data were filtered using fastp version 0.23.2 [31] and were assembled by metaWRAP version 1.3.2 [32]. The selection parameters for metagenome-assembled genomes (MAGs) were set at >50% completeness and <10% contamination. Then, all MAGs were annotated by GTDB-tk version 2.4.0 [33]. CoverM version 0.7.0 [34] was used to calculate the abundance differences in the *Fusobacterium* genus between healthy individuals and cholera patients. Additionally, the abundance of the *Fusobacterium nucleatum* CNBGCC 1850029 across multiple cohorts was evaluated based on 16S rRNA sequence analysis (parameter: --min-read-aligned-percent 80 --min-read-percent-identity 99 --min-read-aligned-length 80). Cohort-related information is presented in Supplementary Table S1. The codes for relative abundance analysis from cohort-based studies are openly available in Zenodo (ID: 17150950).

### 2.2. Bacterial Strains and Growth Conditions

*V. cholerae* C6706 colonies grown on Luria–Bertani (LB) broth with 1.5% (m/v) agar (Cat: A8190, Solarbio Life Sciences Co., Ltd., Beijing, China) were resuspended in LB broth (Cat: HB9305, Qingdao Haibo Biotechnology Co., Ltd., Qingdao, China), and the optical density at 600 nm (OD_600_) was adjusted to approximately 1.0. This suspension was diluted 1:100 in 2× LB broth containing 10 mM HEPES (4-(2-Hydroxyethyl) piperazine-1-ethanesulfonic acid) (Cat: C0215, Beyotime Biotechnology Co., Ltd., Shanghai, China) and then mixed at a 1:1 ratio with 0.22 µm membrane (Cat: FF372, Beyotime Biotechnology Co., Ltd., China) filter-sterilized culture collected from *F. nucleatum* CNBGCC 1850029. A 200 µL aliquot of the mixture was transferred to a 96-well plate (Cat: 3599, Corning, New York, NY, USA). Bacterial growth was monitored by measuring the OD_600_ every 30 min for 24 h using a Spark microplate reader (Spark, Tecan, Männedorf, Switzerland) under aerobic conditions. Separately, *F. nucleatum* strain CNBGCC 1850029 was cultured anaerobically in BGI104 medium (Cat# BGICC_Medium001, Beijing Genomics Institute, Shenzhen, China) within an atmosphere of 85% N_2_, 5% CO_2_, and 10% H_2_.

### 2.3. Coaggregation Assay

Bacteria were washed with PBS and resuspended in anaerobic aggregation buffer (150 mM NaCl, 1 mM Tris/HCl, 0.1 mM CaCl_2_, and 0.1 mM MgCl_2_, pH = 7) at OD_600_ = 1.5. Equal volumes (0.5 mL) of each bacterium were added to tubes, vortex mixed for 10 s, and read at OD_600_ (0 h). Samples were incubated for 1 h at 37 °C anaerobically, and OD_600_ was recorded (1 h). Aggregation was calculated using the following equation: 100% − ((OD_600_ at 0 h − OD_600_ at 1 h) × 100%) [12].

### 2.4. Bacterial Growth Curves and Biofilm Formation Assays

Monocultures of *F. nucleatum* CNBGCC 1850029 and *V. cholerae* C6706 were used for biofilm formation assays. After 48 h of anaerobic growth, the culture from *F. nucleatum* was collected, filtered through a 0.22 µm membrane, adjusted to pH 7.4, and mixed with or without 2X BGI104 broth at a 1:1 ratio. *V. cholerae* was then inoculated at a 1:1000 dilution with 10 mM HEPES. Biofilms were formed by static incubation at 37 °C for 24 h under aerobic conditions [7]. In addition, *F. nucleatum* and *V. cholerae* were co-cultured in BGI104 medium at a 1:1 ratio for 48 h for dual-species biofilm formation under anaerobic conditions. The cell suspension was removed, and the biofilms adhering to the tube walls were rinsed three times with PBS (Cat: E607008, Sangon Biotech (Shanghai) Co., Ltd., Shanghai, China). Subsequently, the biofilms were stained with crystal violet (Cat: G1062, Solarbio Life Sciences Co., Ltd., Beijing, China), resolved by DMSO (Cat: 30072418, Sinopharm Chemical Reagent Co., Ltd., Shanghai, China) and their density was quantified using a microplate reader (Spark, Tecan, Männedorf, Switzerland) at an absorbance wavelength of 570 nm.

### 2.5. Gene Expression of vpsT

*F. nucleatum* CNBGCC 1850029 and *V. cholerae* C6706were co-cultured at a 1:1 inoculation ratio and a 1:100 dilution in a trans-well system, with *F. nucleatum* in the upper chamber and *V. cholerae* in the lower chamber. Subsequently, the *V. cholerae* were collected by centrifugation under the following conditions: 12,000 rpm, 2 min, 4 °C. Then, RNA for real-time PCR (RT-PCR) was extracted using an RNA extraction kit. The RT-PCR reaction was performed using SYBR green fluorescent dye and the CFX Connect Real-time Detection System. The gene recA (recA-RT-F:3′-5′ GTGCTGTGGATGTCATCGTTGTTG; recA-RT-R:3′-5′ CCACCACTTCTTCGC CTTCTTTGA) was used for normalization. Relative expression levels were calculated using the 2^−∆∆CT^ method. For reverse transcription, approximately 400 ng of RNA was extracted using the HiScript II 1st Strand cDNA Synthesis Kit. The primers of vpsT (VCA0952) for RT-PCR as follows: vpsT-RT-F: 5′-GAGTTACCGGCACGATAATG-3′; vpsT-RT-R: 5′-ACTGTCCGCAGGATATTG-3′.

The plasmid pBBR-Lux was linearized using restriction enzymes *BamH*I (Cat: R3136S New England Biolabs (Beijing) Ltd., Beijing, China). The sequence of the *vpsT* promoter was amplified by PCR using FastPfu DNA Polymerase (Cat: AP221, TransGen Biotech Co., Ltd., Beijing, China) (vpsT-pro-F: 5′-CTCACTATAGGGCGAATTGGAGCTCCTGCTTTCAAGGTGAAGTG-3′; vpsT-pro-R: 5′-TTTTGCGGCCGCAACTAGAGGATCGCATGCAAACATCAGAAAG C-3′). The purified PCR products were recovered using the GeneJET gel extraction kit (Cat: 0692, Thermo Scientific, Waltham, MA, USA), and the linearized vector was assembled using Gibson Assembly^®^ Cloning Kit (Cat: E5510S, New England Biolabs (Beijing) Ltd., Beijing, China) according to the manufacturer’s instructions. The resulting construct was transformed into *E. coli* DH5α (Cat: CD201, TransGen Biotech Co., Ltd., Beijing, China). Positive clones were screened by PCR (pBBRlux-Check-F: 5′-TTCCATTCGCCATTCAGG-3′; pBBRlux-Check-R: 5′-TGTA TGTCCTGCGTCTTG-3′) and verified by sequencing. The confirmed plasmids were then introduced into *V. cholerae* C6706 via electroporation. To prepare electrocompetent cells, *V. cholerae* was washed twice with ice-cold 0.02 mM CaCl_2_ and once with 10% glycerol.

The *V. cholerae* with plasmid P*vpsT*-Lux were incubated overnight at 37 °C for 12 h. The culture from *F. nucleatum* CNBGCC 1850029 was collected according to the report above, then mixed with 2× LB broth at a 1:1 ratio. *V. cholerae* was then inoculated at a 1:100 dilution with 10 mM HEPES and challenged with 1% bile salt (Cat: T4009, MERCK, Darmstadt, Germany). Subsequently, 200 µL was transferred to a 96-well plate to measure the growth curve using a microplate reader combined with luminescence detection. Readouts were performed every 30 min for 3 h.

### 2.6. Competitive Colonization Dynamics of V. cholerae and F. nucleatum in the Gut of Adult Mice

A competitive colonization assay between *V. cholerae* C6706 and *F. nucleatum* CNBGCC 1850029 was performed in adult ICR female mice (4 weeks old, approximately 20 g in body weight, Jiangsu GemPharmatech Co., Ltd., Nanjing, China). *F. nucleatum* was rendered streptomycin-resistant via mutagenesis before administration by oral gavage [35]. Mice were pretreated with sterile water containing streptomycin (5 g/L) (Cat: A610494, Sangon Biotech (Shanghai) Co., Ltd., Shanghai, China) and sucralose (0.02 g/L) (Cat: A420074, Sangon Biotech (Shanghai) Co., Ltd., Shanghai, China) for 24 h. *V. cholerae* and *F. nucleatum* were recovered from −80 °C stocks by streaking on selective agar plates and incubated overnight or for 48 h. Fresh cultures were harvested, washed, and adjusted to OD_600_ = 1.0. For co-infection, *V. cholerae* and *F. nucleatum* were mixed at a 1:1 ratio, pelleted by centrifugation (8000 rpm, 2 min), and resuspended in anaerobic 2% NaHCO_3_. Monocultures of *V. cholerae* were processed similarly as controls. Bacterial counts of *V. cholerae* were quantified by plate counting prior to inoculation. Mice were fasted for 8 h and administered 100 μL of 10% NaHCO_3_ to neutralize gastric acid 15 min before oral gavage with 100 μL inoculum (5 × 10^8^ CFU/mice; control group received *V. cholerae* alone, *n* = 5/group). Fecal samples were collected on days 1, 3, 5, and 7 post-inoculation, homogenized with 2 mm steel beads (60 Hz, 120 s), serially diluted, and plated on LB agar supplemented with streptomycin and X-gal. Colony-forming units (CFU) per mg of feces were calculated to assess bacterial colonization dynamics [36]. All animal procedures were approved by the Institutional Animal Care and Use Committee of Huazhong University of Science and Technology.

### 2.7. Bacterial Quantification

The quantitative PCR (qPCR) reactions were performed in a final volume of 25 μL, containing 2 μL of template DNA (50 ng), 12.5 μL of SYBR Green Master Mix (Cat: 11201ES50, Yeasen Biotechnology (Shanghai) Co., Ltd., Shanghai, China), 10 μL of sterile, nuclease-free water, and 0.25 μL each of forward and reverse primers at a concentration of 10 μM. A standard curve was established to correlate the bacterial loads with qPCR detection (CFX connect real-time detection system, Bio-Rad, Hercules, CA, USA). DNA was extracted by QIAamp Fast DNA Stool Mini Kit (Cat: 51604, QIAGEN, Venlo, The Netherlands). *F. nucleatum* cultures were serially diluted, and the CFU/mL was determined. The logarithm of the CFU/mL values was then correlated with the measured Ct values using linear regression. The universal 16S rRNA gene-targeted primers used were as follows: Forward: 5′-TGGAGCATGTGGTTTAATTCGA-3′, Reverse: 5′-TGCGGGACTTAACCCAACA-3′ [37]. For *F. nucleatum* CNBGCC 1850029-specific detection, the following primers were employed: Forward: 5′-CAACCATTACTTTAACTCTACCATGTTCA-3′, Reverse: 5′-GTTGACTTTACAGAAGGAGATTATGTAAAAATC-3′ [12]. The thermal cycling protocol consisted of an initial denaturation at 95 °C for 3 min, followed by 39 cycles of amplification. Each cycle included denaturation at 95 °C for 10 s, annealing at 55 °C for 30 s, and a final dissociation step of 95 °C for 10 s, 65 °C for 5 s, and 95 °C for 5 s. The relative abundance of *F. nucleatum* was calculated as its colony-forming unit (CFU) count divided by the total bacterial load, represented by the 16S rRNA gene quantity.

### 2.8. Small-Molecule Extraction

To extract small molecules from *F. nucleatum* CNBGCC 1850029 metabolic supernatants, bacterial cultures were first inoculated in BGI104 medium and incubated anaerobically at 37 °C for 48 h. Following incubation, cells were pelleted by centrifugation (8000× *g*, 10 min), and the cell-free supernatant was collected. Ethyl acetate (Cat: 10009418, Sinopharm Chemical Reagent Co., Ltd., Shanghai, China) was added to the supernatant at a 1:1 (*v*/*v*) ratio, followed by vortexing for 5 min and sequential phase separation steps: the mixture was allowed to settle for 10 min, vigorously mixed again, and settled for an additional 10 min to maximize extraction efficiency. The organic phase was then isolated, dried, and stored at −20 °C until further use [38]. Parallel negative controls were processed identically using uninoculated BGI104 medium. Prior to experimentation, dried extracts were reconstituted in LB broth, filtered sequentially through 0.22 μm membranes to remove insoluble particles and ensure sterility, and adjusted to pH 7.4. Extracts were standardized to a 2× relative concentration to approximate physiological metabolite levels in bacterial supernatants. Heat inactivation was performed by incubation in a 100 °C water bath for 10 min. All experimental steps included corresponding uninoculated medium controls to validate specificity and exclude background interference.

### 2.9. Untargeted Metabolomics (LC-MS)

Metabolomic profiling of the ethyl acetate extracts was performed by Sanshu Biotech Co., Ltd. (Shanghai, China). The samples were added 200 μL 30% acetonitrile solution (Cat:360457, MERCK, Darmstadt, Germany) to be re-dissolved, homogenized, and centrifuged for 15 min at 14,000 rpm at 4 °C; afterwards, the supernatant was taken for computer detection [39]. All solvents were of LC-MS grade. And ultra-pure water in-house prepared using a Milli-Q water purification system (Millipore, Bedford, MA, USA). The sample extracts were analyzed using a UPLC-Orbitrap-MS system (UPLC, Vanquish; MS, HFX) (Thermo Fisher Scientific, Waltham, MA, USA). UPLC conditions: The analytical conditions were as follows, UPLC: column, Waters HSS T3 (100 × 2.1 mm, 1.8 μm); column temperature, 40 °C; flow rate, 0.3 mL/min; injection volume, 2 μL; solvent system, water (0.1% acetic acid, Cat:45754, MERCK, Darmstadt, Germany): acetonitrile (0.1% acetic acid); gradient program, 0 min phase A/phase B (100:0, *v*/*v*), 1 min phase A/phase B (100:0, *v*/*v*), 4 min phase A/phase B (40:60, *v*/*v*), 6.5 min phase A/phase B (5:95,*v*/*v*), 6.6 min phase A/phase B (100:0, *v*/*v*), 8.0 min phase A/phase B (100:0, *v*/*v*). MS conditions: HRMS data were recorded on a Q Exactive HFX Hybrid Quadrupole Orbitrap mass spectrometer equipped with a heated ESI source (Thermo Fisher Scientific, Waltham, MA, USA) utilizing the Full-ms-ddMS2/MS acquisition methods. The ESI source parameters were set as follows: spray voltage, −2.8 kV/+3.0 kV; sheath gas pressure, 40 arb; aux gas pressure, 10 arb; sweep gas pressure, 0 arb; capillary temperature, 320 °C; and aux gas heater temperature, 350 °C.

The obtained metabolites were annotated using Kyoto Encyclopedia of Genes and Genomes, followed by a quantitative analysis of the metabolites. The candidate compounds were purchased from Macklin Inc., Shanghai, China, and dissolved using DMSO solvent. Final concentrations of 100 μg/mL, 10 μg/mL, and 1 μg/mL were applied to evaluate their effects on the biofilm formation of *V*. *cholerae* in LB medium.

### 2.10. Statistical Analysis

The results are reported as means with their respective standard errors. The results were analyzed using a two-tailed Student’s *t*-test and One-way analysis of variance (ANOVA). Statistical analysis was performed using the software GraphPad Prism v.9 for Windows, and *p* < 0.05 was considered to indicate statistical significance. All experiments were conducted with three independent replicate experiments.

## 3. Results

### 3.1. The Abundance of Fusobacterium Genus in the Cholera Cohorts

**Figure 1:**
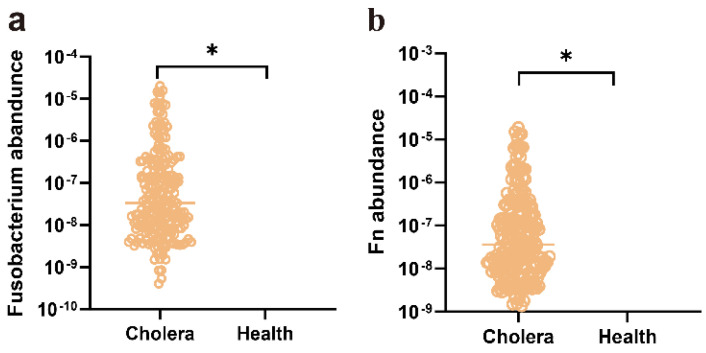
Differential abundance of *Fusobacterium* in the *c*holera cohorts. (**a**) The six publicly available gut metagenomic datasets from cohort-based studies on *c*holera were accessed in the NCBI database. They were divided into a healthy control group and a cholera group. The raw data were analyzed by fastp version 0.23.2, metaWRAP version 1.3.2, and GTDB-tk version 2.4.0. The coverM software (version 0.7.0) was used to calculate the abundance differences in the *Fusobacterium* genus between healthy individuals and cholera patients. (**b**) The abundance of the *Fusobacterium nucleatum* CNBGCC 1850029 across multiple cohorts was evaluated based on 16S rRNA sequence analysis. Fn, *F. nucleatum* CNBGCC 1850029. Significance was determined by two-tailed Student’s *t*-test; *p*-values: ns, not significant, *, *p* < 0.05.

Studies in cetaceans revealed a gradient of decreasing microbial richness from the oral cavity to the hindgut. Concomitantly, the community composition shifted, with *Fusobacterium*-dominated niches in the oral cavity and esophagus transitioning to *Vibrio*-enriched assemblages in the gastrointestinal tract, which included the presence of *V*. *cholerae* [40]. Both *F*. *nucleatum* and *V*. *cholerae* are pathobionts residing in the human gut [4,26]. Their spatial co-localization provides an opportunity for coexistence and interaction. To investigate the role of the *Fusobacterium* genus in the cholera cohorts, we analyzed six publicly available gut metagenomic datasets from cohort-based studies on cholera obtained from the NCBI database. The significant difference was observed in the abundance of the *Fusobacterium* genus between fecal samples from healthy controls and cholera patients (Figure 1a, Supplementary Table S1).

In the *Fusobacterium* genus from the cholera cohorts, *F*. *nucleatum* strain CNBGCC 1850029 was isolated from our laboratory’s intestinal microbial bank, and other *Fusobacterium* strains were not present. Subsequently, to investigate the abundance of *F. nucleatum* CNBGCC 1850029 at the strain level within a cholera cohort, the coverM software (version 0.7.0) was employed for further analysis. In the cholera patients, the results showed *Fusobacterium* was present in a number of patients (n = 220), while it was undetectable in others (n = 504) (Figure 1a). Interestingly, *F. nucleatum* CNBGCC 1850029 was statistically significant compared to the healthy controls (Figure 1b). The underlying reason for this observation remains unknown.

### 3.2. The Supernatant of F. nucleatum Inhibits the Growth of V. cholerae but Promotes Its Biofilm Formation

**Figure 2:**
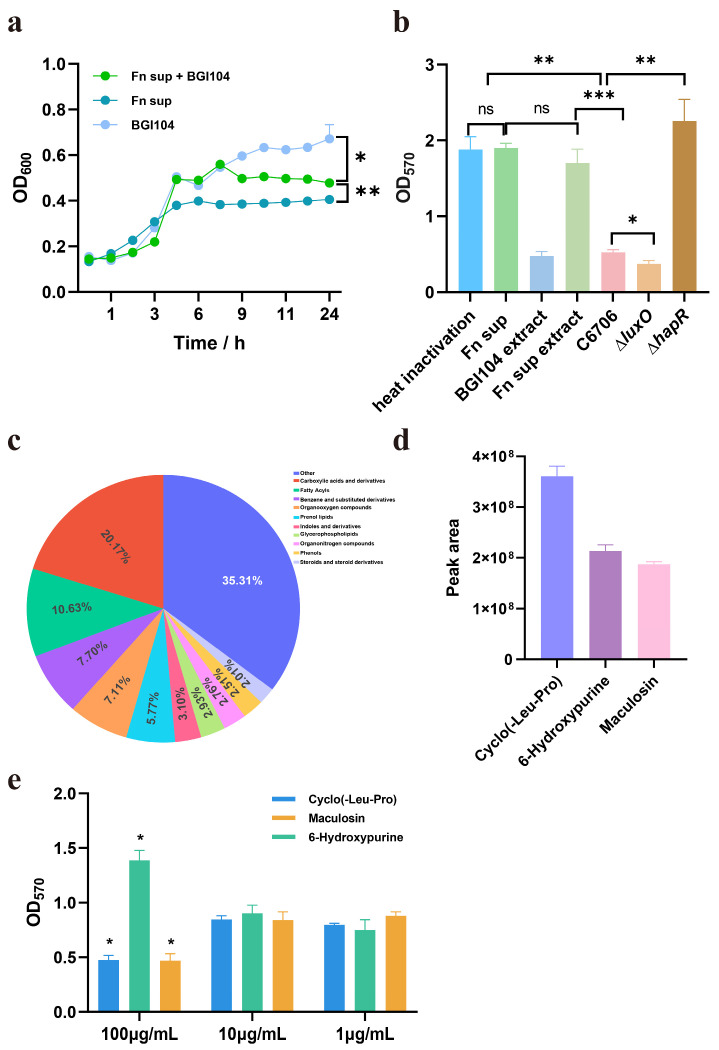
*F. nucleatum* supernatant inhibits the growth of *V. cholerae* but promotes biofilm formation. (**a**) Growth curve of *V. cholerae* with cell-free supernatant of *F. nucleatum*. (**b**) The metabolized supernatant by *F. nucleatum* with pH-neutralized (7.2) affects the biofilm formation of *V. cholerae*. (**c**) Composition of metabolites. An untargeted metabolomic analysis was performed on metabolites extracted from *F. nucleatum* CNBGCC 1850029 using ethyl acetate. The pie chart shows the distribution of the identified metabolite classes. (**d**) Top-ranked metabolites. Cyclo (Leu-Pro), 6-hypoxanthine, and maculosin were identified as the three most abundant metabolites. (**e**) Biofilm modulation by candidate metabolites. The impact of 6-hypoxanthine, cyclo (Leu-Pro), and maculosin, selected based on the metabolomic analysis on *V. cholerae* biofilm formation was assessed. Vc: *V. cholerae* C6706. Fn: *F. nucleatum* CNBGCC 1850029. Significance was determined by *t*-test; *p*-values: ns, not significant, *, *p* < 0.05, **, *p* < 0.01, ***, *p* < 0.001.

To investigate why *F. nucleatum* strain CNBGCC 1850029 is present in the feces of cholera patients and whether it interacts with *V. cholerae*, we first examined the effect of *F. nucleatum* CNBGCC 1850029 on *V. cholerae* C6706 growth. We cultured *V. cholerae* overnight in pH-neutralized (7.2), cell-free supernatant from a 24 h *F. nucleatum* CNBGCC 1850029 culture with or without 2X BGI104 medium. The results showed that compared to BGI104 medium alone, the growth of *V. cholerae* was significantly inhibited after 24 h in the supernatant of *F. nucleatum* CNBGCC 1850029 (Figure 2a). When cultured in a mixed medium containing both the supernatant of *F. nucleatum* CNBGCC 1850029 and BGI104, growth was significantly restored. However, it still differed significantly from that in BGI104 medium alone (Figure 2a). The underlying mechanism remains to be elucidated (Figure 2a).

To investigate whether metabolic interactions between *F. nucleatum* and *V. cholerae* modulate biofilm formation in *V. cholerae*, *V. cholerae* was incubated with the processed supernatant. Using ∆*luxO* as the negative control and ∆*hapR* as the positive control, the results demonstrated that compared to the wild-type (WT), ∆*luxO* exhibited a significant reduction in biofilm formation, while ∆*hapR* showed a significant increase, which is consistent with previous reports [41]. In addition, a marked enhancement in biofilm formation was observed (Figure 2b). When the culture supernatant of *F. nucleatum* was heat-killed, it also increased the biofilm formation of *V. cholerae* (Figure 2b). To further characterize the bioactive components, we extracted *F. nucleatum* supernatant with ethyl acetate. Intriguingly, the ethyl acetate extracts similarly promoted biofilm formation in *V. cholerae* (Figure 2b). These results indicate that *F. nucleatum* metabolites promote *V. cholerae* biofilm formation, independent of growth and high-temperature conditions.

To further identify the specific metabolites involved, an untargeted metabolomic analysis was performed. The results indicated that these metabolites were primarily enriched in categories such as carboxylic acids and derivatives, fatty acyls, benzene and substituted derivatives, and organooxygen compounds (Figure 2c). The top three most abundant metabolites derived from *F. nucleatum* CNBGCC 1850029 were identified as cyclo (Leu-Pro), 6-hypoxanthine, and maculosin (Figure 2d). Further investigation revealed that 6-Hypoxanthine at a concentration of 100 µM enhanced biofilm formation in *V. cholerae*, while no significant effect was observed at other concentrations tested (Figure 2e). In the marine bacterium *Halomonas titanicae* KHS3, hypoxanthine can also promote biofilm formation [42]. However, both cyclo (Leu-Pro) and maculosin at a concentration of 100 µM inhibited biofilm formation in *V. cholerae*. These results confirm and extend prior literature reporting anti-biofilm functionality. For instance, cyclo (Leu-Pro) produced by *Lactiplantibacillus plantarum* CCFM8724 inhibits the growth of *Streptococcus mutans* and *Candida albicans* in mixed-species biofilm formation [43]. The cyclic dipeptide cyclo (Leu-Pro) produced by marine *Bacillus amyloliquefaciens* mitigates biofilm formation and virulence in *Listeria monocytogenes* [44]. Additionally, maculosin can inhibit biofilm formation in *Pseudomonas aeruginosa* PAO1 through quorum-sensing inhibition [45]. In conclusion, these findings underscore the complexity of the metabolic interaction between *F. nucleatum* CNBGCC 1850029 and *V. cholerae* C6706 and necessitate further investigation into targetable metabolites.

### 3.3. F. nucleatum and V. cholerae Co-Aggregate to Form Biofilms by Activating vpsT

**Figure 3:**
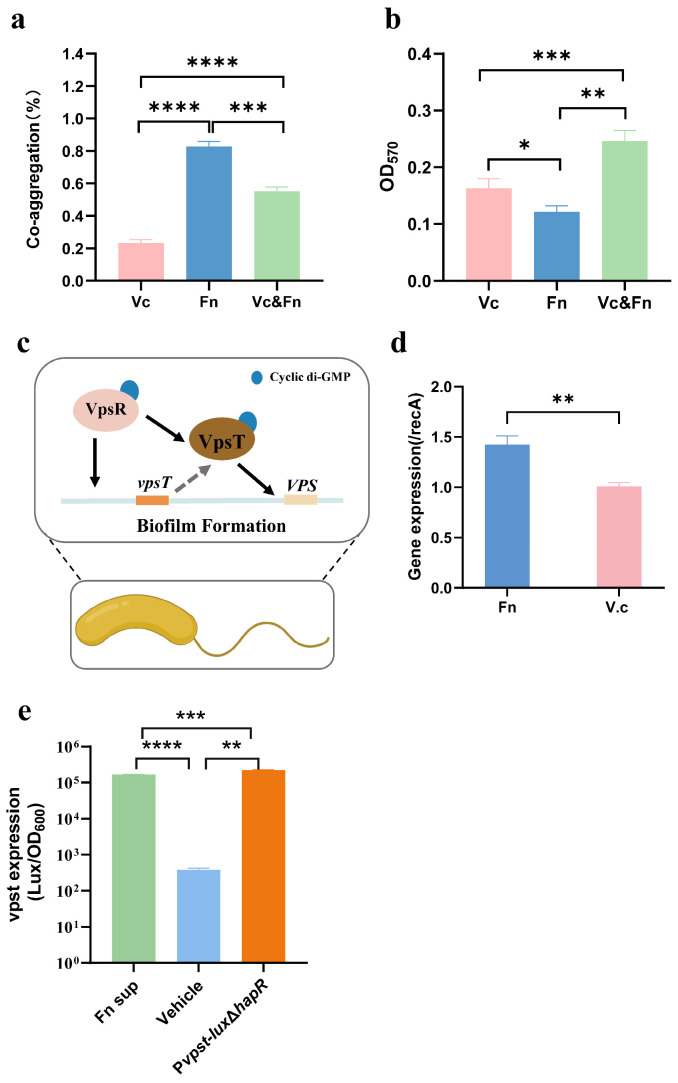
Co-aggregation and subsequent biofilm formation by *F. nucleatum* and *V. cholerae* operate via a VpsT-dependent mechanism. (**a**) aggregation. (**b**) biofilm formation between *F. nucleatum* and *V. cholerae*. (**c**) biofilm formation in *V. cholerae* is governed by a regulatory circuit in which VpsR and VpsT, both activated by the intracellular signal cyclic di-GMP, function as key regulators. Specifically, VpsT directly promotes the transcription of the VPS (Vibrio Polysaccharide) operons, ultimately driving the synthesis of the exopolysaccharide matrix required for biofilm assembly. (**d**) Expression of the *vpsT* gene was assessed using a trans-well system. (**e**) Lux reporter assays. The plasmid P*vpsT*-Lux was electro-transferred into the *V. cholerae* strain. OD_600_ and LuxCDABE were detected, and relative light units were normalized by OD_600_. Different colors indicate distinct genes, proteins, samples, or substances. Solid lines show the direction of the cascade reaction, while dashed lines represent the translation of genes into proteins. Vc: *V. cholerae* C6706. Fn: *F. nucleatum* CNBGCC1850029. Significance was determined by *t*-test; *p*-values: *, *p* < 0.01, **, *p* < 0.01, ***, *p* < 0.001, ****, *p* < 0.0001.

Coaggregation serves as a bridge for biofilm formation [9]. *F. nucleatum*, a bacterial species commonly recognized for its coaggregation capabilities, has not been previously investigated for its ability to form co-aggregates with *V. cholerae*. To evaluate whether their interaction involves coaggregation, we conducted in vitro coaggregation assays. The results demonstrated that while both *F. nucleatum* CNBGCC 1850029 and *V. cholerae* C6706 possess an intrinsic ability to auto-aggregate, their dual-species co-inoculation significantly enhanced interbacterial coaggregation compared to mono-species cultures (Figure 3a). Further co-culture of *F. nucleatum* CNBGCC 1850029 and *V. cholerae* C6706 significantly enhanced dual-species biofilm formation compared to their respective mono-species biofilms (Figure 3b).

In *V. cholerae*, biofilm regulation involves transcriptional activators (e.g., VpsR, VpsT, AphA), repressors (e.g., HapR, H-NS), alternative sigma factors (RpoN, RpoS, RpoE), small regulatory RNAs, and signaling molecules [46]. As VpsT is a master regulator of biofilm formation [47] (Figure 3c), we utilized a trans-well system and lux reporter assays to confirm that its expression was significantly upregulated (Figure 3d,e). These findings collectively demonstrate that the supernatant of *F. nucleatum* CNBGCC1850029 enhances biofilm formation by activating *vpsT*-mediated pathways.

### 3.4. F. nucleatum Promotes the Adaptability of V. cholerae in the Gut of Adult Mice

**Figure 4:**
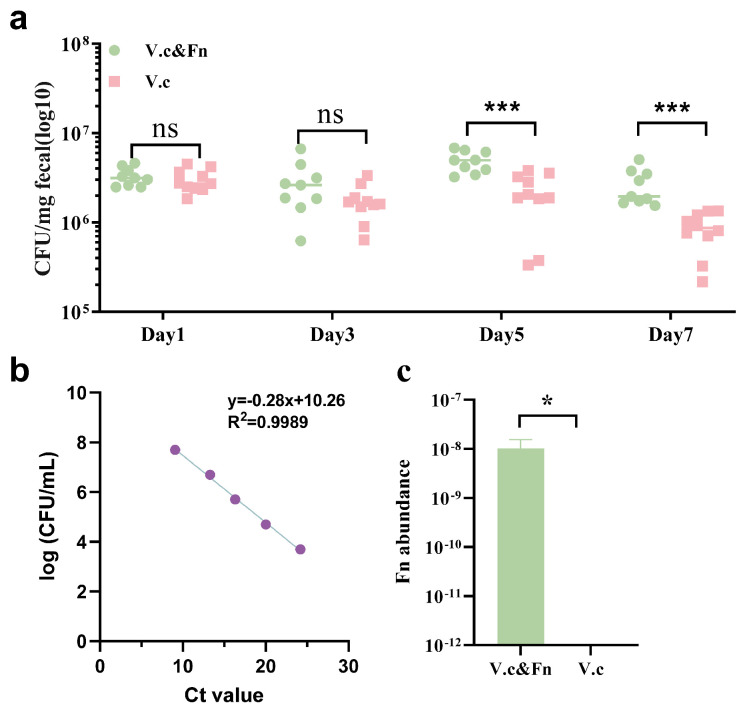
*F. nucleatum* promotes the adaptability of *V. cholerae* in adult mouse model. (**a**) The CFU values of *V. cholerae* post inoculation were shown. Adult mice were infected with *F. nucleatum* and *V. cholerae* at a ratio of 1:1 (*n* = 9) or *V. cholerae* alone (*n* = 10). Numbers of cultivable *V. cholerae* (CFU) recovered from mono-infection or co-garaged mice feces were calculated with standardization based on fecal weight and the input CFU. (**b**) A standard curve was established to correlate the bacterial load with qPCR detection. DNA was extracted from *F. nucleatum* cultures following serial dilution and precise enumeration of CFU/mL. The logarithm of the CFU/mL values was then correlated with the measured Ct values using linear regression. (**c**) *F. nucleatum* abundance or total bacterial load in fecal samples was determined by using real-time quantitative PCR. V.c: *V. cholerae* C6706. Fn: *F. nucleatum* CNBGCC1850029. Significance was determined by *t*-test; *p*-values: ns, not significant; *, *p* < 0.05; ***, *p* < 0.001.

To investigate the role of *F. nucleatum* CNBGCC1850029 in the biofilm formation of *V. cholerae* C6706 in vivo, an adult mouse model was used with streptomycin treatment to test for the colonization of *V. cholerae*. It is worth noting that the adult mouse model has been established to study the mechanisms of *V. cholerae* in response to various intestinal stress environments, and it has been widely used by many groups [35,48]. Compared to infant mice, adult mice have a fully developed human immune system, Reactive Oxygen Species (ROS), and a mature gut microbiota, which provide advantages in studying the mechanisms by which *V. cholerae* responds to host stress during infection [48]. The intestinal adaptability of *V. cholerae* was evaluated by enumerating cultivatable bacteria from collected fecal pellets. After treatment with streptomycin for 24 h, the mice were inoculated with either *V. cholerae* alone or *V. cholerae* and *F. nucleatum* (SmR) at a 1:1 mixture (approximately 10^9^ CFU) by oral gavage. The CFU counts of the *V. cholerae* recovered from the feces at day1 and day3 postinfection were not obviously different between the single and mixed species groups. At day5 and day7 postinfection, the *V. cholerae* number in the feces of mice infected with only *V. cholerae* was more than 3-fold higher than that in the feces of mice infected with the *V. cholerae* and *F. nucleatum* mixture (Figure 4a). For the quantitative assessment of *F. nucleatum*, a linear regression model was generated to define the relationship between the Ct values and the logarithmic CFU/mL (Figure 4b). At day7, the abundance of *F. nucleatum* in the feces of the co-infection group was about 10^−8^. However, *F. nucleatum* was not detected in the group administered *V. cholerae* alone (Figure 4c). These results suggest that coinfection with *F. nucleatum* helps the biofilm formation of *V. cholerae*, which can enhance its adaptability in the gut of adult mice.

## 4. Discussion

Based on the analysis of a cholera cohort and our microbial bank, *F. nucleatum* CNBGCC1850029 was found to be significantly more abundant in cholera patients than in healthy controls. Functionally, although *F. nucleatum* CNBGCC1850029 metabolites inhibited the growth of *V. cholerae* C6706, they promoted biofilm formation. Untargeted metabolomic analysis revealed that *F. nucleatum*-derived metabolites, specifically 6-hypoxanthine, enhance biofilm formation in *V. cholerae*. Furthermore, *F. nucleatum* enhanced bacterial coaggregation with *V. cholerae* and upregulated the biofilm-associated regulatory gene *vpsT*. These findings were further validated in an adult mouse model, confirming that *F. nucleatum* enhances the gut adaptability of *V. cholerae*, thereby elucidating a mechanism for their co-existence during infection.

Host-derived intestinal metabolites and bacterial proteins intricately regulate the formation and dispersal of *V. cholerae* biofilms. Primary bile salts, such as taurocholate, glycocholate, and deoxycholate, promote the dispersal of *V. cholerae* biofilms, thereby facilitating intestinal colonization [49]. Also, indole, a tryptophan degradation product that acts as a bacterial signaling molecule, may also modulate biofilm dynamics [50]. Additionally, exogenous *para*-aminobenzoic acid (pABA) is utilized in folate biosynthesis, which reduces bacterial stress and upregulates the expression of fimbrial adhesins, thereby enhancing bacterial colonization. However, pABA simultaneously suppresses extracellular polysaccharide production and attenuates virulence in vivo [51]. Collectively, these metabolites and proteins exert synergistic or antagonistic effects to shape the biofilm lifecycle and pathogenic progression of *V. cholerae*.

In this study, the extracts of *F. nucleatum* metabolites by ethyl acetate significantly promoted biofilm formation in *V. cholerae*. Untargeted metabolomic analysis revealed that 6-hypoxanthine enhances biofilm formation in *V. cholerae*, although the supernatant of *F. nucleatum* inhibits the growth of *V. cholerae*. This suggests that 6-hypoxanthine may function as a co-aggregation promoter. Beyond 6-hypoxanthine, this study verifies that metabolites like cyclo (Leu-Pro) and maculosin, commonly known for their antibacterial properties, can act as inhibitors of biofilm formation [43,44,45]. The presence of such antimicrobial substances in the metabolome might contribute to the observed growth inhibition of *V*. *cholerae*, although this hypothesis requires substantial experimental validation.

In polymicrobial communities, the close coexistence of different species significantly amplifies their interactions. These interactions include nutrient sharing, quorum sensing (QS), and coordinated gene expression [52]. It is precisely these complex dynamics that shape the community’s overall architecture, enhance its ability to evade host immune attacks, and lead to persistent, difficult-to-eradicate chronic infections, posing serious challenges for clinical treatment [24,25]. Therefore, researchers must not only decipher the mechanisms by which dual- or multi-species biofilms resist environmental stress but also develop advanced methodologies to track the dynamic evolution of species within these communities [53]. A key limitation of the present study is the lack of omics-based validation or novel tracking methods to analyze the composition and dynamic changes in the co-aggregated biofilm community. As reported in the literature, it is possible to successfully track all species within a nine-member mixed biofilm over 72 h [54]. Further research should employ such innovative methods to dissect the species dynamics in co-aggregation-derived biofilms. In the future, the work should also employ transposon mutant library screening to identify which *F. nucleatum* adhesins interact with which receptors on *V. cholerae* to scaffold biofilm formation [55,56].

Bacterial aggregation leads to the formation of multicellular aggregates enclosed by an extracellular matrix composed of polysaccharides, proteins, lipids, and DNA, ultimately resulting in a biofilm. This enhances the environmental adaptability of bacteria and enables their persistent survival across different ecological niches [57]. The synthesis of biofilm components in *V. cholerae* is primarily regulated by VpsT and VpsR. Proteins such as HapR, H-NS, AphA, and VqmA are also involved and interact with each other, forming a complex regulatory network. Additionally, small RNAs in *V. cholerae* participate in the regulation of biofilm development by degrading or stabilizing the mRNA of target genes [58]. To more comprehensively investigate the impact of the microbe-free supernatant of *F. nucleatum* CNBGCC1850029 on *V. cholerae* C6706 biofilm formation, transcriptomics should be employed to obtain gene transcription profiles, followed by verification.

Establishing effective infection models for *V. cholerae* remains a significant challenge, as existing animal models fail to fully replicate human infection conditions. After streptomycin treatment of the adult mouse intestine, the diversity and structure of the gut microbiota are disrupted, thereby freeing up ecological niches and enabling *F. nucleatum* CNBGCC1850029 and *V. cholerae* C6706 to interact within the murine intestinal environment [35]. In this model, antibiotic treatment disrupts the diversity and structure of the gut microbiota, thereby simplifying the interaction between *F. nucleatum* CNBGCC1850029 and *V. cholerae*. In reality, the gut microbiota serves as a critical factor in responding to pathogenic infections, and *V. cholerae* inevitably interacts with a wide range of intestinal bacteria beyond *F. nucleatum* CNBGCC1850029 [3,4]. Future research should therefore account for the influence of the gut microbiota on the interaction between *F. nucleatum* CNBGCC1850029 and *V. cholerae* C6706, employing multi-omics strategies to elucidate the underlying molecular mechanisms and colonization dynamics of their interplay.

## 5. Conclusions

In sum, this study reveals a “collaborative pathogenesis” model, in which *F. nucleatum* facilitates the intestinal adaptation of *V. cholerae*, thereby providing a theoretical foundation for understanding the infection dynamics of this pathogen.

## Data Availability

The data presented in this study are openly available in Biomarkers analysis from Cholera cohort at https://doi.org/10.5281/zenodo.17150950, reference number [17150950].

## Supplementary Materials

The following supporting information can be downloaded at https://www.mdpi.com/article/10.3390/microorganisms14010211/s1, Supplementary Table S1: Species abundance table; Supplementary Table S2: Analysis of ethyl acetate extracts of *F. nucleatum* CNBGCC1850029 metabolites.
